# Rebound in prevalence and intensity of *Onchocerca volvulus* infection five years after cessation of alternative treatment strategies in the Massangam Health District, West Region, Cameroon: need for coordinated and sustained efforts

**DOI:** 10.1371/journal.pntd.0013849

**Published:** 2025-12-22

**Authors:** Gabriella S. Ondoua Nganjou, Laurentine Sumo, Arnauld Efon Ekangouo, Narcisse Nzune Toche, Jean Bopda, Jeanne C. Sondi Dissake, Ivana Youmbi Kammogne, André Domche, Yannick Niamsi Emalio, Théophile M. Mpaba Minkat, Georges B. Nko’Ayissi, Shannon M. Hedtke, Warwick N. Grant, Flobert Njiokou, Joseph Kamgno, Hugues C. Nana Djeunga

**Affiliations:** 1 Translational Research and Development Foundation (TREND Foundation), Yaoundé, Cameroon; 2 Higher Institute for Scientific and Medical Research (ISM), Yaoundé, Cameroon; 3 Department of Animal Biology and Physiology, Faculty of Science, University of Yaoundé I, Yaoundé, Cameroon; 4 Department of Animal Biology and Physiology, Faculty of Science, University of Ebolowa, Ebolowa, Cameroon; 5 Department of Biochemistry, Faculty of Science, University of Yaoundé I, Yaoundé, Cameroon; 6 Department of Public Health, Faculty of Medicine and Biomedical Sciences, University of Yaoundé I, Yaoundé Cameroon; 7 Ministry of Public Health, Yaoundé Cameroon; 8 Department of Environment and Genetics, La Trobe University, Melbourne, Victoria, Australia; Seoul National University College of Medicine, KOREA, REPUBLIC OF

## Abstract

**Background:**

The control of onchocerciasis currently relies on yearly distribution of ivermectin to at-risk populations. To tackle onchocerciasis in areas of high transmission (so-called hotspots) and achieve elimination of transmission, several complementary/alternative strategies (biannual ivermectin, doxycycline-based test-and-treat and vector control) were implemented in Massangam Health District (HD) in Cameroon, following World Health Organization (WHO) guidelines. A short-term impact evaluation revealed significant reductions in the endemicity levels in three focal hotspot communities (Makouopsap, Mankakoun, and Njinja-Njingouet). This study aimed to assess the situation of onchocerciasis in the focal hotspot communities five years after the cessation of the short pilot implementation of Alternative Treatment Strategies (ATS).

**Methodology:**

A quantitative cross-sectional survey was conducted in December 2023 in three focal hotspot communities of the Massangam Health District. Participants underwent a comprehensive assessment including parasitological examination (skin snipping) to establish *Onchocerca volvulus* microfilaridermia.

**Principal findings:**

The overall prevalence of *O. volvulus* infection in the three focal communities was 18.7% (95% confidence interval CI: 14.1-24.3), significantly higher in the Mankakoun (30.8%) and Makouopsap (24.0%) communities compared to the Njinja-Njingouet community (7.0%) (p = 0.001). The intensity of infection was 3.136 (standard deviation, SD: 19.3099) mf/ss, ranging from 5.218 mf/ss (Mankakoun community) to 2.840 mf/ss (Njinja-Njingouet community). Parasitological indicators significantly increased five years after the cessation of ATS in all three focal communities (p = 0.0409).

**Conclusion/Significance:**

These findings indicate a rebound in onchocerciasis transmission and underscore the need for coordinated and sustained efforts, implemented at the scale of a transmission zone per WHO recommendations, to achieve elimination goals.

## Author summary

Despite significant progress in controlling onchocerciasis through mass drug administration (MDA) with ivermectin, transmission persists in certain context despite uninterrupted efforts. To accelerate the elimination of infection, a package of alternative and complementary treatment strategies (ATS) was piloted in an area of higher transmission of onchocerciasis (Massangam Health District, West Region, Cameroon), resulting in a significant decrease in disease burden. We conducted a cross-sectional survey in three sentinel communities of the Massangam Health District five years after cessation of ATS. Our findings revealed a rebound of *Onchocerca volvulus* infection in the sentinel communities, suggesting a local rebound in infection consecutive to the fact that the area was yet to be cleared from infection, and/or the reintroduction of parasites from neighbouring health districts where such elimination effort (ATS) was not implemented. These findings underscore the urgent need for sustained (as long as needed to reach interruption of transmission indicators) and coordinated (in a transmission zone rather than at health district level) control efforts, especially those with proven impact such as test-and-treat with doxycycline or semi-annual ivermectin mass distribution, to achieve the WHO’s elimination goals.

## Introduction

Onchocerciasis, also known as river blindness, is a disease caused by infection with the parasitic nematode *Onchocerca volvulus* that is transmitted to humans through the bites of its blackfly vectors, which breed in fast-flowing rivers and streams. Female *O. volvulus* produce microfilariae (mf) that migrate to the skin, where they are responsible for the cutaneous clinical manifestations of onchocerciasis [1,2]. These microfilariae can also penetrate the eyes, leading to inflammatory lesions (keratitis, chorioretinitis), optic nerve atrophy, and total blindness [1]. Onchocerciasis may also lead to neurological disorders, including epilepsy [3], nodding syndrome [4], and growth retardation [5], thus representing a major public health burden in sub-Saharan Africa. An estimated 21 million people suffer from the disease, with around 99% of cases reported in 31 sub-Saharan African countries, including Cameroon [6].

Ivermectin treatment administered to all eligible persons at risk, under community guidelines, is the major World Health Organization (WHO) recommended intervention in most endemic countries [7]. This strategy has had significant impacts on onchocerciasis endemicity and transmission in several epidemiologically favorable settings. Indeed, the WHO verified the elimination of parasite transmission in Colombia, Ecuador, Mexico, and Guatemala in 2013, 2014, 2015, and 2016 respectively, and declared these four countries free from the disease [8]. Additionally, in some endemic countries in Africa such as Mali, Senegal, and Nigeria, the transmission of the disease has been interrupted by ivermectin alone in some foci [9,10], leading to the shift in the paradigm of the fight against onchocerciasis from disease control to elimination of transmission [2,6]. However, in Africa, despite these successes and several decades of regular mass drug administration (MDA), the transmission of onchocerciasis persists in certain contexts [11–13].

Ivermectin significantly reduces *O. volvulus* microfilaridermia to levels below that required for efficient transmission by blackflies (microfilaricidal effect). This drug also has a proven temporary suppressive effect on microfilarial production by adult female worms (embryostatic effect), and a deleterious – though moderate - impact on the adult worm viability (macrofilaricidal effect), especially when repeatedly administered [2]. Since ivermectin is not fully adulticidal and adult worms have a long lifespan (10–15 years), control efforts relying on annual distribution of ivermectin need to be sustained for 15–20 years (or even longer) if one wants to interrupt the transmission of the infection [14,15]. Therefore, to accelerate the elimination of onchocerciasis, WHO recommends alternative treatment strategies (ATS) where transmission persists despite the recommended annual treatment with ivermectin (area of higher transmission so-called hotspots) [16]. In Cameroon, several areas of higher transmission have been identified, including in the Massangam Health District, West Region, where very high transmission indicators have been described despite over two decades of uninterrupted annual distribution of ivermectin (IVM) with satisfying coverage rates (≥80%). To accelerate transmission interruption in this focus, a comprehensive set of ATS was implemented in 2017 and 2018 in three local communities identified as drivers of onchocerciasis transmission at the district-scale: Makouopsap, Mankakoun, and Njinja-Njingouet [17]. The implemented ATS consisted of two rounds of test-and-treat using doxycycline, biannual Community-Directed Treatment with ivermectin (CDTI) in communities surrounding the hotspot focal communities, and ground larviciding vector control upstream of breeding sites on River Mbam (2) and Nja (3) closest in proximity to focal communities. These efforts reduced the prevalence and intensity of the *O. volvulus* infection by more than 50% in each hotspot focal community [18].

**Fig 1:**
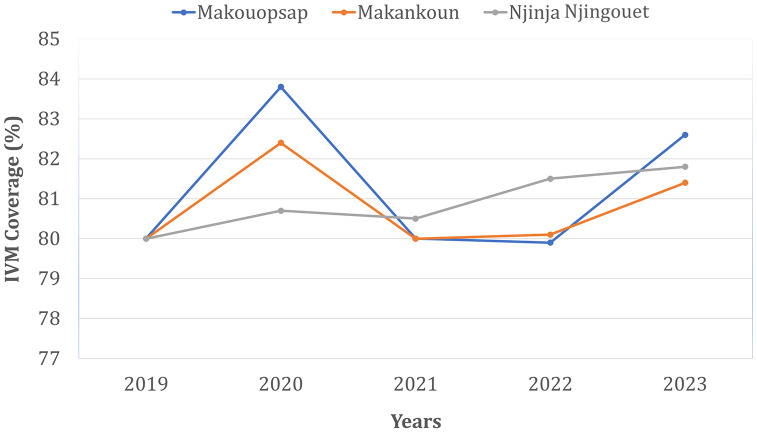
Reported therapeutic coverages in the Massangam health district. Data were gathered on ESPEN website [19]. The red line represents the cut-off treatment coverage recommended by WHO.

The ATS was a pilot study that stopped in 2018. Routine yearly CDTI then resumed in the entire Health District, with reported treatment coverage higher than 80% (Fig 1). This study aimed to assess prevalence and intensity of *O. volvulus* infection in the hotspot communities of the Massangam Health District five years after the cessation of ATS, to inform strategic decisions and reach the WHO 2030 goal [6].

## Methods

### Ethics statement

Prior to the commencement of this study, ethical clearance was granted by the West Regional Ethics Committee of Research for Human Health (CRERSH-Ou) (N°1009/ 29/ 11/ 2023/ CE/ CRERSH-OU/ VP), and administrative authorizations were obtained from Regional Delegation for Public Health (DRSP) of the West Region (N°1112/ L/ MINSANTE/ DRSPO/ PNLMTN) and the Massangam District Medical Officer. After clear explanations of the pertinence of the study to all potential participants, written informed consents, assents, or parental consents were obtained from all volunteers, their parents or their legal guardians. The privacy of all participants was safeguarded, including confidentiality and anonymity during data collection, processing, and reporting by assigning a unique identifier to each participant.

### Study area and selection of sites

The study was conducted in the Massangam Health District, located in the West Region of Cameroon. It is a rugged and mountainous terrain, with peaks reaching impressive heights of up to 3,000 meters above sea level. These geographical features contribute to the presence of numerous fast-flowing rivers, which create conditions suitable for the breeding and survival of blackflies, and thus facilitate continuous transmission of onchocerciasis in the area [17,18]. The Massangam Health District is crossed by two main rivers, the Noun and the Mbam, which lie to the southwest and the east parts of the Health District respectively, as well as their tributaries, the Nja and Kim rivers. The population of this Health District is estimated at approximately 23,067 inhabitants [20]. Additionally, Massangam Health District shares boundaries with other Health Districts where onchocerciasis is highly endemic, such as Bafia, Ntui, and Ndikinimeki, creating a network of interconnected areas affected by the disease.

**Fig 2:**
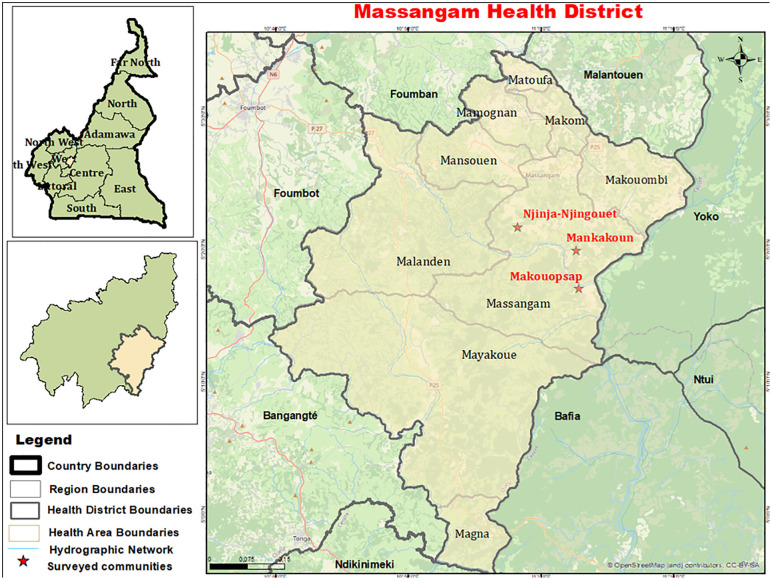
Map of the study area showing the focal communities (area of higher transmission) in the Massangam Health District. The base layer of the map is freely available on OpenStreetMap (https://www.openstreetmap.org/#map=12/5.3521/10.9093) under the Open Database License (ODbL) 1.0) compatible with CC BY 4.0.

Three specific communities (Makouopsap, Mankakoun, and Njinja-Njingouet) (Fig 2) were purposively selected for the present study as they served as study sites (focal communities) during the implementation of alternative strategies to accelerate onchocerciasis elimination in the Massangam Health District. They are firstline communities, i.e., are located <5 km from the river where there are accessible blackfly breeding sites [17,18], and they belong to the ~ 12 km radius of high transmission defined during entomological and epidemiological surveys in 2015 and 2016 [2]. In 2016, the infection in these communities was 45.8% (95% CI: 34.0-58.0) in Makouopsap, 38.3 prevalence % (95% CI: 26.1-51.8) in Mankakoun, and 22.4% (95% CI: 13.1-34.2) in Njinja-Njingouet prior to the implementation of ATS in 2017 and 2018 by the National Onchocerciasis Control Program (NOCP) and its partner, Sightsavers [2,18]. Following the implementation of ATS, the infection prevalence drastically decreased in the three focal communities to 15.7% (95% CI: 9.2-24.2), 18.7% (95% CI: 12.2-26.7), and 1.0% (95% CI: 0.2-5.5) in Makouopsap, Mankakoun, and Njinja-Njingouet, respectively [18].

### Study design and enrolment of participants

A quantitative cross-sectional survey was conducted in December 2023 in the three focal communities (areas of higher transmission) of the Massangam Health District (Makouopsap, Mankakoun, and Njinja-Njingouet), approximately 9 months after the last CDTI round. All individuals, both male or female, aged 5 years and above, and permanent residents or those who had already lived for at least 5 years in the selected communities were eligible for the study.

All eligible individuals were given the opportunity to participate. In each selected community, a house-to-house sensitization was conducted to inform, mobilize and invite population to a strategic location (the community chiefdom or health centre) for the interview and clinical examination. Prior to their enrolment, the objectives and procedures of the study were clearly explained to potential participants and those who agreed to participate provided written informed consents or assents; parental consents were obtained from parents, caregivers, or legal guardians of minors. Socio-anthropological information (name, age, sex, treatment history, reasons for non-compliance to treatment) were recorded using a structured questionnaire (S1 Text). Anonymity was guaranteed for each participant by a unique identifier and a barcode for accurate tracking. Electronic forms were developed using ODK Collect and deployed on a tablet; data were collected in real time and stored on a web server (ona.ia).

### Skin snip examination

Skin snip samples were collected for the parasitological diagnosis of onchocerciasis. Briefly, the skin was cleaned with an antiseptic solution (70% ethanol) and two skin biopsies (one from each posterior iliac crest) were collected using a sterile 2mm corneoscleral Holth-type punch. A sample of approximately 7 mm^2^ (2 mg) of each piece of skin taken was incubated at room temperature for 24 hours in saline solution. The incubation solution was then pipetted on a slide and examined using bright-field microscopy at the magnitude 100x. All the microfilariae that have emerged from the skin snips were identified and enumerated, and the intensity of infection expressed as the number of microfilariae per skin snip (mf/ss).

### Statistical analyses

All the relevant data - socio-anthropological (socio-demographic data, history and compliance to CDTI, duration of residence in the community) and parasitological (presence and number of *O. volvulus* microfilariae) - were downloaded from the ONA server in Microsoft Excel format, then imported to IBM SPSS version 26 (SPSS Inc., Chicago, IL, USA) for statistical analyses. Categorical variables, including the CDTI coverage and infection with *O. volvulus*, were expressed as the percentage of persons who have taken ivermectin for the past 10 years or infected individuals among the total number examined. Age was stratified into classes (59; 10–14; 15–20; 21–49 and ≥50 years old) for categorical analysis. Continuous variables, including microfilarial densities, were expressed as arithmetic means with a 95% confidence interval (95% CI). Intensity of infection was also measured by the community microfilarial load (CMFL), estimated as the geometric mean of the number of microfilariae per skin snip (mf/ss) among adults aged ≥ 20 years in a community, using the log (x + 1) transformation to account for zero counts, which are particularly common after several rounds of ivermectin treatments.

Prevalence of infection with *O. volvulus* and/or proportions of participants who have taken ivermectin mass treatment (CDTI) for the past 10 years and those never treated were compared between sexes, communities, and age groups using Pearson’s Chi-Square, with correction for continuity, or Fisher’s exact test when appropriate. Intensities of infection were compared between covariates using the Mann-Whitney and Kruskal-Wallis tests. The difference between the prevalence of *O. volvulus* infection when the ATS were stopped [18] and 5 years after cessation of ATS (present study) was evaluated using the Chi-square test for paired samples. For all statistical analyses, the level for significance was set at 5%.

## Results

A total of 225 individuals aged 5–102 years (Median: 28 years; IQR: 11–46) were enrolled in the three focal communities of the Massangam Health District. The sex ratio was significantly male-biased (0.73). A substantial proportion of enrolled individuals (41.0%) refused to participate in the skin snip examination because of “painful” procedure.

### History of—and adherence to—ivermectin mass treatments

Overall, 53.3% (95% CI: 46.8-59.7) of the participants reported having taken ivermectin 1–3 times, 12.9% (95% CI: 9.1-17.9) 4–6 times, and 33.8% (95% CI: 27.9-40.2) 7–10 times during the past 10 years (2014–2023), with no significant difference between sexes (Chi-square = 2.092; df = 2; p = 0.351). Similarly, there was no significant difference in compliance to ivermectin treatments among different communities (Chi-square = 8.991; df = 4; p = 0.061), although Makouopsap had the highest recorded rate of individuals who declared having taken ivermectin for the past 10 years (44.4%), while Mankakoun had the lowest one (17.3%). The proportion of individuals who acknowledged having taken ivermectin mass treatments for the past 10 years significantly increased with age: adults exhibited the highest compliance rates (40.0% for individuals aged 21–49 years and 20.9% for ≥50 years) compared to their younger counterparts (6.2% among those aged 15–20 years, 16.0% among those aged 10–14 years, and 16.9% among those aged 5–9 years) (Chi-square = 108.857; df = 8; p < 0.0001).

The proportion of never treated or individuals who never ingested ivermectin tablets during the past 10 years was estimated at 29.9% (95% CI: 24.2-36.1). The reasons reported by the never treated were failure of the drug distributor to deliver ivermectin to the household (48.8%; 95% CI: 34.6-63.2), refusal due to fear of adverse events (including serious adverse events, SAEs) (32.6%; 95% CI: 20.5-47.5), and absence of individuals in the communities during treatment rounds (18.6%; 95% CI: 9.7-32.6).

### Prevalence and intensity of *O. volvulus* infection

**Fig 3:**
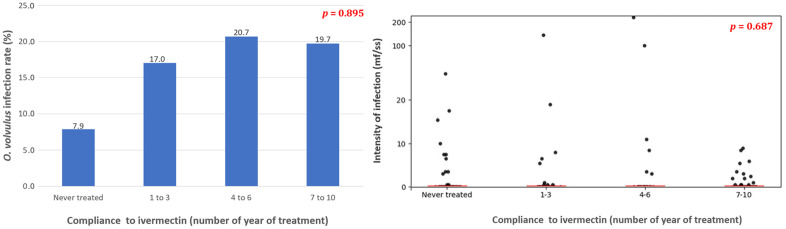
Association between ivermectin treatment compliance and prevalence and intensity of *O. volvulus* infection in the Massangam Health District.

The overall prevalence of *O. volvulus* microfilaridermia in the Massangam Health District was 18.8% (95% CI: 13.8-24.1), significantly higher in the Mankakoun community (30.8%; 95% CI: 18.6-46.4) and Makouopsap community (24.0%; 95% CI:16.7-33.2) compared to the Njinja-Njingouet community (7.0%; 95% CI:3.2-14.4) (Chi-Square = 13.48; df = 2; p = 0.001). Although different among communities, this prevalence was similar between age groups (Chi-Square = 7.317; df = 4; p = 0.120) and sexes (Chi-Square = 2.689; df = 2; p = 0.101) (Table 1). No significant association was found between individual microfilaridermia prevalence and the number of ivermectin doses taken during the last 10 rounds (Chi-Square = 0.221; df = 2; p = 0.895) (Fig 3).

**Table 1:** *Onchocerca volvulus* microfilarial prevalence according to focal communities, sexes, and age groups.

Variables	No. individuals Examined	No. individuals infected (%)	95% CI of infection rate
**Focal communities**			
Makouopsap	100	24 (24.0)	16.7-33.2
Mankakoun	39	12 (30.8)	18.6-46.4
Njinja-Njingouet	86	6 (7.0)	3.2-14.2
**Sex**			
Male	95	29 (13.7)	8.2-22.0
Female	130	13 (22.3)	16.0-30.2
**Age groups**			
5-9	38	2 (5.3)	1.5-17.3
10-14	36	5 (13.9)	5.8-27.3
15-20	14	3 (21.4)	7.6-47.6
21-49	90	20 (22.2)	14.9-31.8
≥50	47	12 (25.5)	15.3-39.5
**Total**	**225**	**42 (18.7)**	**14.1-24.3**

***No** number of;* ***mf:*** *Microfilariae****; CI:*** *confidence interval.*

**Fig 4:**
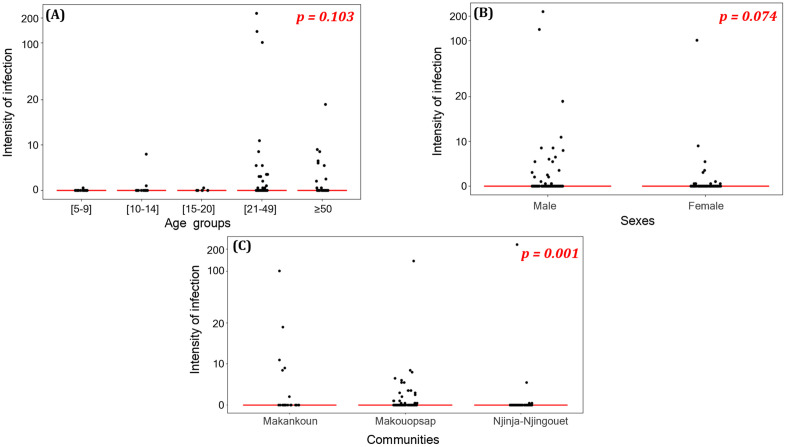
Scatter plots of intensities of infection according to age groups, sexes, and focal communities of the Massangam Health District.

Similar to the prevalence, the intensity of *O. volvulus* infection in the focal communities was 3.136 (standard deviation, SD: 19.3099) mf/ss, significantly higher in the Mankakoun community (Mean: 5.218; SD: 16.6041) compared to the Makouopsap community (Mean: 2.585; SD: 14.4590) and the Njinja-Njingouet community (Mean: 2.840; SD: 25.5560) (Chi-Square = 14.308; df: 2; p = 0.001). The microfilarial density was evenly distributed among age groups (Chi-square = 7.701; p = 0.103) and sexes (MannWhitney = 1.788; p = 0.074) (Fig 4). The overall community microfilarial load (CMFL) in the study area was 0.409 mf/ss (Mankakoun: 1.034 mf/ss, Makouopsap: 0.449 mf/ss, Njinja-Njingouet: 0.138 mf/ss) \(Table 2). The arithmetic mean number of microfilariae per skin snip was not significantly correlated with the intake of ivermectin treatment (r = 0.032; p = 0.687) (Fig 3).

**Table 2:** Intensity of *Onchocerca volvulus* infection according to focal communities, sexes, and age groups.

Variables	No. individuals examined	Microfilarial density (mf/ss)		CMLF
		Mean (SD)	Range	
**Focal communities**				
Makouopsap	100	2.585 (14.4590)	0.0-137.5	0.449
Mankakoun	39	5.218 (16.6041)	0.0-101.0	1.034
Njinja-Njingouet	86	2.840 (25.5560)	0.0-230.0	0.138
**Sex**				
Male	95	4.354 (23.7619)	0.0-230.0	0.403
Female	130	1.468 (10.4318)	0.0-101.0	0.390
**Age groups**				
5-9	38	0.092 (0.4912)	0.0-3.0	0.018
10-14	36	0.958 (3.3581)	0.0-17.5	0.0
15-20	14	3.393 (11.5811)	0.0-43.5	0.0
21-49	90	6.067 (29.8591)	0.0-230.0	0.373
≥ 50	47	1.574 (3.6579)	0.0-19.0	0.588
**Total**	**225**	**3.136 (19.3099)**	**0.0-230.0**	**0.401**

***No*** *number of;* ***mf:*** *microfilariae;* ***ss:*** *skin snip;* ***SD:*** *standard deviation;* ***CMFL:*** *community microfilarial load.*

### Trends in *Onchocerca volvulus* infection in Massangam Health District

**Fig 5:**
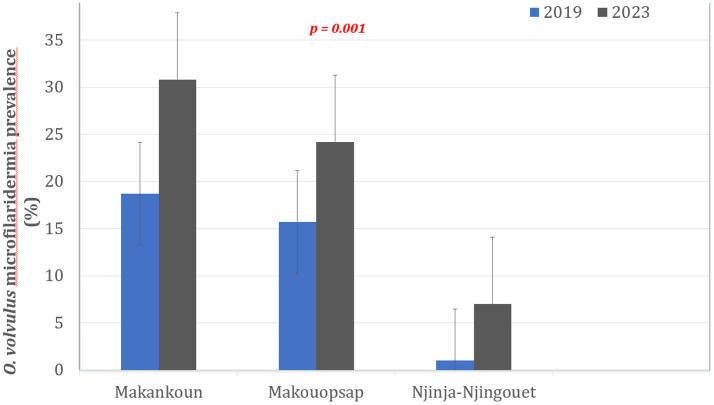
Trends in *Onchocerca volvulus* prevalence at the end of ATS implementation (2019) [18] and five years after cessation of ATS (2023) in the three focal communities of Massangam Health District.

From 2018 (when ATS were stopped) to 2023 (5 years post-stoppage of ATS), the prevalence of onchocerciasis increased from 15.7% to 24.0% in Makouopsap (Chi-square = 1.80; df = 1; p = 0.1797), 18.7% to 30.8% in Mankakoun (Chi-square = 1.88; df = 1; p = 0.1703), and 1.0% to 7.0% in Njinja-Njingouet (Chi-square = 3.01; df = 1; p = 0.0828). These data show that the mf parasitological indicators had increased significantly five years after the cessation of ATS in all three focal communities (18.7%; 95% CI: 14.1-24.3) compared with their baseline levels (at end of ATS implementation) (12.3%; 95% CI: 9.0-16.4) (Chi-square = 4.18; df = 1; p = 0.04) (Fig 5) (S1 Table).

## Discussion

Alternative treatment strategies (ATS) carried out in 2017 and 2018 in three focal communities (Makouopsap, Mankakoun, and Njinja-Njingouet) of the Massangam Health District led to substantial reductions in the prevalence and intensity of *O. volvulus* infection, demonstrating the feasibility of accelerating onchocerciasis elimination even in transmission hotspots for using strategies complementary to CDTI [2,18]. However, this significant success can be jeopardized by (i) the unsustainability of the implementation of the ATS (this was a pilot investment by the Ministry of Public Health and Sightsavers that ran for only two years), and (ii) the proximity to neighboring high-transmission areas that may provide a source for reintroduction of parasites to the Massangam Health District, particularly the Mbam Valley in the Centre Region of Cameroon [11,18]. To test these hypotheses and assess the long-term impact of ATS implemented in the Massangam Health District, we conducted a cross-sectional survey five years after the cessation of alternative/complementary strategies in the three focal communities.

Compared with endline assessment data (collected just at the end of ATS) [18], a marked rebound in the prevalence and intensity of *Onchocerca volvulus* microfilaridermia was observed in the three focal communities, despite continuous effective rounds (≥ 80% coverage) of the routine CDTI reported in Massangam Health District after the cessation of ATS (Fig 1) [19]. Although this rebound was overall statistically significant, the increase at community level did not reach significance, likely due to reduced statistical power because of small sample size. The increase in the prevalence and intensity of infection post-cessation of ATS might have been exacerbated by the unplanned interruption of CDTI in 2020 because of COVID-19. For a 1-year interruption of MDA, mathematical modelling predicts a mean delay of 2–3 years in elimination efforts [21]; i.e., if CDTI alone were effective and optimally implemented in the subsequent years, prevalence should have begun to decrease by 2023 when this study was conducted. However, a recent survey demonstrated that the 1-year discontinuation of CDTI in 2020 had no impact on the level of endemicity of onchocerciasis in the Bafia Health District in the neighboring Mbam Valley (Centre Region) [22]. This means that the prevalence was supposed to remain low post-2018 when this study was conducted. Indeed, a recent survey in the Massangam health district two years post-ATS reported a much lower microfilaridermia prevalence (3.7%) [23], suggesting a residual impact of the ATS at a short period (two years) post-cessation of ATS. The rebound to 18.7% in the overall prevalence observed in our survey conducted five years post-ATS, highlights the transient nature of the gains achieved at short-term [18–23] and underscores that without sustained intervention, even successful, intensive strategies only suppress transmission temporarily, and a rapid rebound occurs once the pressure is lifted, reverting to the baseline forces of transmission in the area over the years. Note that the high dispersion of vector populations and frequent human movement from the Mbam Valley onchocerciasis focus - still under routine CDTI alone, despite the very high on-going transmission documented [11,18] - might also explain the rebound in parasitological indicators of onchocerciasis ongoing transmission in the Massangam Health District, raising the need of coordinated actions and efforts.

The therapeutic coverage survey, though prone to memory bias, revealed a substantial proportion of never treated as already reported in this area [24]. This poor participation in the routine CDTI, even if not significantly associated with *O. volvulus* microfilaridermia and intensity of infection, can explain, at least partly, why the parasitological indicators of onchocerciasis infection increased after cessation of ATS in the Massangam Health District. Indeed, these never-treated individuals act as a consistent source of parasites (reservoirs) that maintain or facilitate transmission. Consequently, even with satisfactory reported coverage, the presence of this untreated segment of the population can undermine control efforts and lead to the rebound of infection as described in this study. Among reasons of non-adherence to routine CDTI, the fear of post-ivermectin adverse events (including serious adverse events, SAEs) was reported, and this can explain why people mostly adhere to test-and-treat with doxycycline [25] which is known to have no effect on the *Wolbachia*-free *Loa loa* and therefore not associated with SAEs [26]. To compound the problem of non-compliance, the force-of-infection in the Massangam Health District is high [17], and parasitological and entomological indicators for transmission interruption were yet to be met when ATS was stopped [18].

As predicted by mathematical models [14,15] and reported by several studies in Cameroon and elsewhere, the use of ivermectin alone is insufficient to eliminate onchocerciasis everywhere, especially in areas of higher transmission where the force-of infection is very high. The ATS used in the Massangam Health District included test-and treat with doxycycline, bi-annual administration of ivermectin, and vector control [16] as an “end-game strategy” [18,27] that clearly demonstrated the potential to accelerate elimination of onchocerciasis even in areas where transmission is sustained despite decades of annual CDTI. To prevent rebound of infection (adult worms that survived the ATS slowly resumed the release of microfilariae, new infections from ongoing transmission, and possible reintroduction from neighbouring areas) and therefore maintain progress toward elimination, long-term intervention is needed for onchocerciasis to be controlled and eliminated [6,14,15]. The observed rebound in onchocerciasis endemicity reported in the present study is a clear example of a setback in the fight against onchocerciasis, suggesting that the use of alternative tools to disrupt the transmission of the parasite should be sustained as long as needed to reach interruption of transmission indicators [18,28,29]. A coherent implementation of strategies involving multiple approaches tailored to the specific needs of areas where the infection persists, including alternative/complementary treatment strategies (ATS) [16], is crucial to achieve the goal of disease elimination. National onchocerciasis control programs and their implementing partners should advocate for substantial supports to sustain their efforts and maintain achievements [30].

Despite the proven efficacy and impact of the test-and-treat strategy with doxycycline, notably with its proven macrofilaricidal effect, particularly valuable for hard-to-reach groups like nomadic or semi-nomadic populations, its operational complexities - including a 4–5-week treatment regimen as well as contraindications for children and pregnant women - present major challenges for long-term community-wide implementation [18]. Given these constraints, a semi-annual (twice-a-year) administration of ivermectin would have been a more pragmatic and sustainable strategy to maintain the gains achieved by the ATS, with a more pronounced embryostatic and macrofilaricidal effects compared to annual regimens.

In addition, these efforts should be coordinated with neighboring endemic areas likely belonging to the same transmission zone [2]. Here, we have noted the possibility that transmission between neighboring health districts could be contributing to the observed rebound in prevalence. The delineation of transmission zones using entomological and human movement data is therefore urgent in order to redefine the geographic scale for where and when to initiate and/or stop MDA and potential ATS. Delineating transmission zones should be a prerequisite for decision making to avoid wasting resources, and to preserve the progress and achievements made so far toward the elimination of onchocerciasis.

## Limitations

The main limitation of this study is the reliance on self-reported treatment history, which may be subject to recall bias especially after more than a year, and could account for the discrepancy between the low observed coverage in this study compared to the high reported coverage. In addition, no dedicated effort was done to reach or distinguish specific groups such as nomadic or semi-nomadic populations which may potentially contribute to the observed differences in treatment coverages [31]. The sample size at community level was low and not proportionately distributed despite the sampling effort, because of the substantial refusal rate (41.0%) for skin snip biopsies always regarded as invasive and painful by the populations. Furthermore, the true prevalence and intensity of O. volvulus infection in this study might have been underestimated because of the use of skin snip microscopy, which is known to be less sensitive compared to molecular diagnostics, especially in low endemicity context [31].

## Conclusion

The significant rebound of prevalence and intensity of onchocerciasis in the Massangam Health District occurred five years after cessation of substantial efforts invested through the implementation of ATS. These results highlight the importance of coordinated and sustained interventions with proven impact such this ATS initiative to achieve the WHO elimination goals highlighted in the 2021–2030 roadmap for neglected tropical diseases (NTDs), to end the neglect caused by the continued transmission of NTDs, and to attain the United Nations sustainable development goals [6].

## Supporting information

S1 Text Semi-structured questionnaire used for the collection of socio-anthropological data. (DOCX)

S1 Table Comparison of parasitological indicators of *O. volvulus* infection between 2019 (short-term impact evaluation) and 2023 (follow up) in the three focal communities of the Massangam Health District. (DOCX)
