# Supplementary material for: Rebound in prevalence and intensity of Onchocerca volvulus infection five years after cessation of alternative treatment strategies in the Massangam Health District, West Region, Cameroon: need for coordinated and sustained efforts
Source: PLoS Negl Trop Dis. 2025 Dec 22;19(12):e0013849. doi: 10.1371/journal.pntd.0013849 (PMC12755783; doi:10.1371/journal.pntd.0013849)
Supplement: S1 Text — (DOCX) [file pntd.0013849.s001.docx]

| Bar code |
| --- |

# CASE REPORT FORM

## SECTION 1. SOCIO-ANTHROPOLOGICAL INFORMATION

### GENERAL INFORMATION

Date: |______|______|________| Region: |_____________| Health District: |_____________|

Health Area: |_____________| Community: |_______________| Community Code: |____|____|____|

Order: |_____|_____|_____| ID: |_____|_____|_____|_____|_____|_____| Sex (M/F): |______|

Age: |________| years old Occupation: |___________________________________________________|

### HISTORY OF POPULATION MOVEMENT (MIGRATION)

For how long have you been living in the village? |_______| years (« 0 » if less than 6 months)

Have you moved out of the village during the last 10 years? Yes |_____| No |_____|

- If “Yes”, how often? |____| once a week |____| once a month |____| once a year
- How many communities/villages have you visited? |______|. Please list them, specifying the corresponding Health District (HD) and duration of the stay (« 0 » if less than 6 months).

1) Community: |_____________________| HD: |_____________________| Duration: |_______| years 2) Community: |_____________________| HD: |_____________________| Duration: |_______| years 3) Community: |_____________________| HD: |_____________________| Duration: |_______| years 4) Community: |_____________________| HD: |_____________________| Duration: |_______| years 5) Community: |_____________________| HD: |_____________________| Duration: |_______| years 6) Community: |_____________________| HD: |_____________________| Duration: |_______| years 7) Community: |_____________________| HD: |_____________________| Duration: |_______| years 8) Community: |_____________________| HD: |_____________________| Duration: |_______| years

Reasons of migration: |____________________________________________________________________|

### HISTORY IVERMECTIN (IVM) TREATMENT

Last year of IVM treatment: |_________________| (« 0 » if never treated / never took IVM)

Number of treatments received during the last 10 years: |__________|

Reasons of refusal (if never treated): |___________________________________________

__________________________________________________________________________________________________________________________________________________________________________________|

## SECTION 2. CLINICAL INFORMATION

Visual acuity (distance at which the participant can distinguish 3 fingers)

- Left eye: |_______| m
- Right eye: |_______| m

| Scabies: Yes \|_______\| |  | No \|_______\| |
| --- | --- | --- |
| Pruritus: Yes \|_______\| |  | No \|_______\| |
| Urticaria: Yes \|_______\| |  | No \|_______\| |
| Leopard skin: Yes \|_______\| |  | No \|_______\| |
| Lizard skin: Yes \|_______\| |  | No \|_______\| |

Presence of onchocercal nodules: Yes |_______| No |_______|

- **Number:** |________|
- **Location:** Head: |_____| Shoulder blades: |_____| Costal grill: |_____|

Coccyx: |___| Trochanter: |___| Iliac crest: |___| Knee: |___|

Shin: |___| Foot: |___| Other (specify): |_________________________|

** Collect pictures of the lesions if the participant agrees.*

### NOTES (CLINICAL INFORMATION)

|__________________________________________________________________________________________________________________________________________________________________________________|

## SECTION 3. SAMPLE COLLECTION AND PROCESSING

**Skin Snip collection**

Yes |_______| No |_______|

### Results of skin snips examination and sample storage

- Left Snip: |__________________| mf - Right Snip: |___________________| mf
- Sample storage: Microfilariae (mf): |______| Skin Snips: |______|

**NOTES (SAMPLE COLLECTION AND PROCESSING)**

|__________________________________________________________________________________________________________________________________________________________________________________|
