# Supplementary material for: Rebound in prevalence and intensity of Onchocerca volvulus infection five years after cessation of alternative treatment strategies in the Massangam Health District, West Region, Cameroon: need for coordinated and sustained efforts
Source: PLoS Negl Trop Dis. 2025 Dec 22;19(12):e0013849. doi: 10.1371/journal.pntd.0013849 (PMC12755783; doi:10.1371/journal.pntd.0013849)
Supplement: S1 Table — (DOCX) [file pntd.0013849.s002.docx]

| **Focal communities** | **Post-ATS impact evaluation survey**  **(2019)** | | | | **Current follow-up survey**  **(2023)** | | | | |
| --- | --- | --- | --- | --- | --- | --- | --- | --- | --- |
|  | **No individual examined** | **Mf prevalence**  **(95% CI)** | **GM Mf density**  **(Min, Max)** | **CMFL** | **No individual examined** | **Mf prevalence**  **(95% CI)** | **GM Mf density**  **(Min, Max)** | **CMFL** |  |
| Makouopsap | 102 | 15.7  (9.2 - 24.2) | 3.8  (0, 187) | 0.22 | 100 | 24.0  (16.7-33.2) | 2.585  (0.0, 137.5) | 0.449 |  |
| Mankakoun | 123 | 18.7  (12.2 - 26.7) | 4.5  (0, 243) | 0.49 | 39 | 30.8  (18.6-46.4) | 5.218  (0.0, 101.0) | 1.034 |  |
| Njinja-Njingouet | 99 | 1.0  (0.2 - 5.5) | 2.3 | 0.06 | 86 | 7.0  (3.2-14.2) | 2.840  (0.0, 230.0) | 0.138 |  |
| **Total** | **324** | **15.7**  **(9.2 - 24.2)** | (0, 25) | **0.27** | **225** | **18.8**  **(13.8-24.1)** | **3.136**  **(0.0, 230.0)** | **0.409** |  |

**S1 Table.** Comparison of parasitological indicators of *O. volvulus* infection between 2019 (short-term impact evaluation) and 2023 (follow up) in the three focal communities of the Massangam Health District

*ATS: alternative treatment strategy; Mf: Microfilarial; No: number of; GM: geometric mean; CMFL: community microfilarial load; 95% CI: 95% confidence interval*
